# Erratum: A General Waveguide Circuit Theory

**DOI:** 10.6028/jres.097.024.errata2014

**Published:** 2014-11-10

**Authors:** Dylan F Williams, Roger B Marks

**Affiliations:** National Institute of Standards and Technology, Boulder, CO 80305

J. Res. Natl. Inst. Stand. Technol., Volume 97, Number 5, September-October 1992, p. 533 http://dx.doi.org/10.6028/jres.097.024

**Erratum:** Equations D5 and E3 in the appendices of [1] contain typographical errors. They should read

$$W\equiv \text{diag}(W_{n});W_{n}\equiv \frac{{\tilde{p}}_{on}}{v_{on}\;i_{on}}=\frac{v_{on}^{*}}{v_{on}}\frac{{\tilde{p}}_{on}}{p_{on}^{*}}$$ (D5)

and

U≡diag(|vom|vomRe(Zrefm)|Zrefm|). (E3)

Also, the reference to Eq. (20) just after equation (D5) should be a reference to Eq. (17).

